# Opaque Social Instruments: A Cultural Evolutionary Approach to Pleistocene Symbolic Artifacts

**DOI:** 10.1002/evan.70036

**Published:** 2026-06-22

**Authors:** Corijn van Mazijk

**Affiliations:** ^1^ Faculty of Philosophy University of Groningen Groningen Netherlands

## Abstract

Prehistoric “symbolic” artifacts remain incompletely explained by semiotic models, which emphasize representational meaning but offer limited insight into how such materials emerged and spread across Pleistocene populations. This article develops a cultural evolutionary framework that reconceives early ornaments, pigments, figurines, and related materials as opaque social instruments (OSIs): culturally transmitted, socially functional traits whose coordinating effects typically exceed users' explicit understanding of their roles. A heuristic distinction is introduced between participation‐embedded OSIs, whose efficacy derives from embodied participation in shared, interactionally dense practices, and frame‐embedded OSIs, whose efficacy depends more heavily on publicly stabilized interpretive frames and role structures that extend across contexts and generations. Because these modes operate under different transmission environments and selective pressures, they are expected to generate distinct patterns of reproduction, contextual embedding, and archeological visibility. Reframing prehistoric “symbolic” materials as OSIs shifts explanatory focus from encoded meaning to social coordination, functional opacity, and transmission dynamics, offering a mechanistic account of how materially mediated practices became reproducible components of Pleistocene social systems.

## 1 Introduction

The presence, character, and evolutionary significance of so‐called “symbolic” behavior in the Pleistocene remain central problems in archeological theory. Accumulating evidence over the past two decades has challenged the view that such practices were exclusive to *Homo sapiens* or tied to a late cognitive “revolution” [1, 2, 3]. Across multiple hominin contexts, archeologists have documented recurrent pigment use and curation [4, 5], apparently overdetermined or display‐inflected lithic forms [6, 7], sustained traditions of personal ornamentation [8, 9], and bodily display [10, 11]. Together, these materials indicate patterned, recurrent behaviors whose persistence is not straightforwardly explained by immediate subsistence or technical demands.

However, this expanding corpus has not yielded corresponding conceptual clarity. What so‐called “symbolic” artifacts are, what functions they served (if any), and how they should be theoretically interpreted remain widely debated. Debates suffer from “loose reference to symbolic culture” ([12], 5) and “lack of definition” [13], providing little guidance on what such symbols amount to cognitively or materially, or how their evolutionary emergence could be identified in the record.

The dominant interpretive vocabulary in Paleolithic archeology has been informed by semiotic models, especially Peircean distinctions between icons, indices, and symbols [14, 15]. Within this framing, “symbolic” artifacts are typically approached as representational signs whose significance depends on convention rather than resemblance or direct causal linkage. In practice, this starting point frequently encouraged an explanatory move from material patterning to questions of encoded meaning, abstract reference, and the cognitive capacities associated with language. While much recent work treats such inferences with appropriate caution, the tendency to equate “symbolic” with representational convention continues to structure debates about when language‐like cognition emerged (e.g. [16, 17, 18, 19]).

The perspective developed here shifts the explanatory focus. Rather than beginning with reference and semantic decoding, it asks how materially mediated practices became socially efficacious, recurrently reproduced, and demographically scalable under recurrent coordination pressures. The central issue is not what prehistoric ornaments, pigments, or figurines represent, but why such material forms emerged and how they were transmitted and integrated into evolving systems of social coordination.

To that end, prehistoric “symbolic” materials are reconceived, in many cases, as opaque social instruments (OSIs): socially consequential objects whose coordinating roles need not have been explicitly grasped by their users, who instead experienced them as beautiful, sacred, customary, or otherwise normatively compelling, without representing the broader coordination dynamics that sustain them. This does not imply that such materials lacked other functions, only that their coordinating roles form an important and partly opaque dimension of their persistence and reproduction. An evolutionary explanation must therefore consider the coordination pressures under which such practices become advantageous, the ways in which they are learned and reproduced under conditions of opacity, and the transmission processes through which they are reproduced, retained, and sometimes extended across populations.

The account that follows is intended as a starting architecture rather than a finished taxonomy. It specifies core claims and transmission logic, while leaving room for future work to refine taxonomies, derive sharper archeological signatures, and integrate additional learning and ecological variables. The discussion proce in three steps. Section 2 revisits dominant semiotic approaches in Paleolithic archeology, and clarifies the limits of treating “symbolic” artifacts primarily as representational signs. Section 3 develops the basic OSI framework as a cultural evolutionary account of functionally opaque, materially mediated coordination practices. Building on this foundation, Section 4 introduces a heuristic distinction between two modes of social embedding: participation‐embedded OSIs (PE‐OSIs) and frame‐embedded OSIs (FE‐OSIs).

Taken together, the present account recasts the category of “symbolic artifacts” as an evolutionary‐social problem rather than a primarily semiotic one, and carries broader cross‐disciplinary implications for understanding how materially mediated coordination practices emerge, become reproducible, and diversify in human cultural evolution. In archaeological contexts, this perspective yields a set of high‐level implications concerning the transmission environments through which such practices become socially efficacious. The present paper does not aim to provide a comprehensive archaeological signature model or exhaustive operationalization, as its primary objective is to outline the conceptual and evolutionary transmission logic through which materially mediated coordination practices can emerge, persist, and diversify under conditions of functional opacity.

## 2 From Signs to Materially Mediated Coordination

Archaeological descriptions of Middle Stone Age beads, pendants, ocher pieces, or engravings as “symbolic” typically inherit a semiotic framework rooted in nineteenth‐ and early twentieth‐century thought. In this lineage, Peirce's icon–index–symbol triad classifies signs according to the kind of sign–object relation involved: icons by resemblance, indices by physical or causal connection, and symbols by a relation that is arbitrary in the specific sense of being neither resemblance‐based nor causally grounded, but sustained through socially stabilized interpretive habits [14, 15]. Importantly, the arbitrariness at issue concerns the sign–object relation, not the randomness of conventions themselves, which are structured and socially maintained. Together with Saussure's signifier–signified model [20], this typology has provided a tacit template for classifying prehistoric artifacts, even where “symbol” is used without explicit theoretical commitment.

In archaeological usage, this framework easily encourages treating Pleistocene “symbolic” materials as instances of convention‐based reference in something like a code‐like, language‐adjacent sense. Although some recent contributions approach such inferences cautiously and emphasize ecological, cognitive, or social contexts (e.g [21]), a substantial portion of the debate still begins by construing such artifacts as representational signs and proceeds from material patterning to questions of encoded meaning, abstract reference, and the cognitive capacities associated with language. The interpretive trajectory is thereby pulled toward a linguistic model: artifacts or engravings become tokens that stand for something absent, and that would be “read” by those who share the relevant interpretive habits (e.g. [22]). This orientation has deeply shaped discussions of “modern behavior,” where beads, pigments, or engravings are frequently treated as proxy evidence for language‐like symbolic cognition (e.g. [16, 18, 23, 24, 25, 26, 27, 28, 29, 30, 31, 32]).

Yet this symbolic framing does not always travel straightforwardly into the Pleistocene. Semiotic categories were developed to analyze sign systems in which arbitrary sign–object relations play a central role. But genuinely arbitrary, code‐like sign systems are rare in deep time. Even writing, emerging only in the last six millennia, initially relied heavily on iconic and substitution strategies before fully arbitrary sign relations stabilized [33, 34]. By contrast, bead strings, pendants, or body pigments may be socially effective without functioning as arbitrary symbols in the strict sense. Ornamental practices can regulate interaction, recognition, and affiliation without encoding syntactically articulable messages. Their efficacy can be enacted rather than decoded: people acquire norm‐guided responses to them through repeated participation in shared activity, not by learning propositional content or “the code” in anything like a linguistic sense.

Even with recent conceptual refinements (e.g [35, 36]), the archaeological utility of Peirce's schema may remain limited for deep‐time contexts. One central difficulty is that the typology groups together phenomena generated by radically different cognitive, developmental, and interactional processes. A thorn bug's camouflage, the Makapansgat pebble reportedly collected by an Australopithecus, and the Lion Man of Hohlenstein‐Stadel all qualify as icons under Peircean criteria, yet the capacities and social contexts underlying these cases differ profoundly [37]. The category of symbol fares no better: even where it might apply, it compresses diverse forms of reference that, in language, depend on distinct cognitive operations, such as displacement, deixis, abstraction, or compositional structuring.

Recent theoretical work in archeology has increasingly criticized internalist, representation‐heavy models of cognition and has instead emphasized how material culture participates in perception, action, and meaning‐making. This shift is continuous with phenomenological and pragmatist approaches that treat meaning as emerging in situated, embodied activity, and is visible across several strands, including Material Engagement Theory [38, 39], new materialist and ontological approaches to images and visual culture (e.g. [40, 41, 42, 43]), practice‐ and activity‐based accounts of Paleolithic visual cultures and ornamentation (e.g. [44, 45]; see also [46, 47, 48]), as well as related 4E‐inspired work in cognitive archeology emphasizing perception–action coupling in skilled material engagement (e.g. [49]). What unites these approaches is not a rejection of meaning, but a rejection of the assumption that “symbolic” significance is best explained by decoding representational content or by treating artifacts as externalized symbols of language‐like cognition.

Yet even as these perspectives relocate meaning within embodied and socially embedded activity, a further question remains underspecified for the Pleistocene record: by what mechanisms do materially mediated practices become copied, reproduced, and distributed across populations? Current representational critiques motivate a shift away from meaning‐decoding toward social embedding, but they do not by themselves specify the transmission dynamics through which such practices emerge and persist across demographic and ecological change. The framework developed here is intended to help clarify this further explanatory level by drawing on cultural evolutionary principles to model how social learning biases, demographic environments, and recurrent coordination pressures shape the emergence, reproduction, and transformation of “symbolic” practices.

This reorientation thus relocates the explanatory focus from symbolic meaning to materially mediated coordination. Instead of asking primarily what an artifact represented or how it would have been decoded, we can ask how certain material forms became recurrent features of coordinated practice, why some were preferentially copied and retained, how they were made learnable and compelling without explicit representation of their social roles, and what interactional and demographic conditions allowed them to persist and diversify.

## 3 A General Account of Opaque Social Instruments (OSIs)

From a cultural evolutionary perspective, tools, practices, and ideas that are reliably reproduced across space and time often do so because they generate advantages within particular transmission ecologies [50, 51, 52]. That is to say: broad, widely replicated cultural patterns can be treated analogously to biological traits at least in this sense: they may persist insofar as they confer particular material, social, or psychological advantages. Over the past decades, psychologists and evolutionary anthropologists have fruitfully applied this line of reasoning to ritual, myth, and religious practices (e.g. [50, 53, 54, 55, 56, 57, 58, 59, 60, 61, 62]). Importantly, from the outside, such practices often appear puzzling or “non‐utilitarian,” and even participants rarely give coherent causal accounts of why they exist. Yet these traditions often prove functional in particular ways that remain opaque to users.

Such functional opacity from the human standpoint is not, then, an anomaly; extensive work on cultural transmission shows that practices can persist and spread even when participants lack causal insight into the processes that sustain them. Prehistoric beadwork, pigment use, ornamentation, and other “symbolic” practices are traditions that endured and expanded over vast temporal scales. As such, they are, at least at first glance, continuous with this broader pattern, even if the mechanisms underlying their long‐term reproduction and persistence remain to be specified.

Within Paleolithic archeology, there is some precursor literature that treats stylistic patterning and display as socially consequential, including classic style/isochrestic approaches (e.g. [63, 64, 65]), later signaling‐oriented models (e.g. [7, 66, 67, 68]), and “cosmetic coalitions” [69, 70]. The OSI framework extends these insights in a different explanatory direction. While some OSIs may involve costly or skill‐based display, the present account does not treat “symbolic” artifacts primarily as signals whose reliability is secured by cost, nor does it center sexual selection or individual quality display as the main explanatory mechanism. Instead, it emphasizes coordination, recognition, and sustained reproduction under conditions of functional opacity. The novelty of the OSI framework lies not in the claim that prehistoric artifacts had partly social functions, but in specifying a basic transmission‐based mechanism through which materially mediated coordination practices can become socially efficacious, reproducible, and archeologically visible.

This is the core claim of this article: so‐called “symbolic” artifacts – ornaments, pigments, figurines, decorated objects, and other materially elaborated forms typically classified as esthetic or non‐utilitarian – can be understood as opaque social instruments (OSIs). This claim has two crucial components.

(1) “Symbolic” objects fulfill diverse social functions

The artifacts at issue are precisely those whose persistence is not adequately explained by immediate instrumental utility alone. Ornaments, pigments, and figurative objects could signal competence, sexual availability, or ritual status; mark lineages or descent groups; stabilize alliances; externalize kinship; buffer group tensions; support exogamy networks; or reinforce narrative memory. As social life grew more complex, investing effort in material forms that helped stabilize identity recognition, affiliation, coordination, and out‐group interactions produced adaptive payoffs.

(2) These functions are typically not consciously represented

Users rarely experience these objects as “tools for cooperation,” “kinship markers,” or “conflict regulators.” Rather, engagement is framed in terms of beauty, sacrality, traditional obligation, or simple custom.

Socially efficacious practices do not require participants to represent the functions they serve. Material traditions can help regulate coordination, affiliation, and social structure without users explicitly understanding these effects. To clarify how that is possible, the following sections identify six families of evolved biases that help explain both the recurrent coordination pressures under which OSI‐like practices become advantageous and the psychological mechanisms through which they are acquired, retained, and reproduced without explicit representation of underlying functions. These are not intended as a complete causal inventory, but as recurring bias families that help explain why OSI‐like practices are both adaptive and reproducible. They can be seen as playing distinct but interacting roles: some (i–iv) primarily illuminate the social conditions that make certain material practices reliable solutions to coordination problems, while others (v–vi) shape how such practices are preferentially copied and retained across generations. The population‐level transmission dynamics that structure their long‐term reproduction and distribution are briefly addressed in the follow‐up section.

### 3.1 Recurrent Coordination Pressures in Human Social Ecologies

(i) Kinship and in‐group favoritism

Humans preferentially invest resources in close kin, with whom they share genes, and they reliably favor in‐group members over out‐groups in trust, empathy, and evaluation, even in pre‐linguistic infancy [55, 71, 72]. Such biases make it difficult to sustain cooperation beyond close kin or primary in‐groups. Material practices can function as OSIs that extend in‐group boundaries, signal shared identity, or modulate kin‐biased tendencies, thereby supporting coordination beyond immediate in‐groups without users needing to articulate this function.

(ii) Coalition and alliance tracking

Humans are exquisitely sensitive to alliance structure [55, 73, 74]. They spontaneously monitor who cooperates with whom and remember information relevant to coalitions without being aware that they do so. In contemporary contexts, phenotypic cues such as racial features can (mis)serve as coalition markers, but for much of prehistory, physiological cues would have been less pronounced. Consequently, material displays like beads and pigments could function as visually salient coalition markers. These can sustain cooperativeness and increase trust, even when people experience them simply as “what one wears.” Such displays may also regulate the affective background of interaction: by reducing uncertainty, tension, or anxiety, they can indirectly stabilize cooperation and affiliation without participants representing this role explicitly.

(iii) Precaution and threat management

Throughout human evolution, intergroup conflict and out‐group threat were constant possibilities, while exogamous exchange and intergroup contact were necessary for gene flow [55, 75, 76, 77]. OSIs can counteract negative out‐group biases by functioning as buffers in otherwise tense social interactions, for example, through gifting, signaling peaceful intent, or indicating shared norms. All the while, users consciously interpret them as customary, prestigious, or sacred adornments.

(iv) Prestige and mate‐choice biases (sexual selection)

Like many species, humans use observable traits as cues to underlying fitness, competence, or status [66, 78]. Prestige and mate‐choice biases make individuals especially attentive to displays that suggest skill, fertility, social status, or access to resources. Ornaments derived from hunting success, craftsmanship, or long‐distance exchange can function as OSIs in sexual competition, even when their users consider them in terms of beauty or tradition.

### 3.2 Why OSIs Can Be Reproduced Without Understanding

(v) Conformity and opaque procedures

Humans display strong conformity and copy group practices with high fidelity, even when no one can explain why a procedure must be followed. This tendency is reinforced by norm psychology and model‐based biases: individuals defer to widely adopted practices, respected models, and socially enforced conventions. Such learning strategies offered selective advantages by accelerating reliable cultural transmission under uncertainty [50, 79]. Ritualized, procedurally opaque actions – whether in food processing, ritual performance, or ornamentation – can therefore persist and spread because deference to socially established practices was evolutionarily advantageous. OSIs can thus be reproduced for generations without participants representing their social‐organizational functions.

(vi) Content, attention, and affective biases in cultural selection

The phenomenal forms that may stabilize are further shaped by systematic biases in attention, memory, and affect, as well as their compatibility with already established “worldviews” [80, 81]. Humans preferentially attend to salient, emotionally charged, distinctive, and/or inferentially rich stimuli, and these features enhance recall and transmission. Minimally counterintuitive elements [54, 56] are another well‐studied case, but symmetry, agentive cues, contrast, and awe‐ or threat‐inducing features likewise increase memorability and cultural retention.

These biases should not be understood as an exhaustive set of discrete adaptive domains. Biases (i–iv) identify recurrent social‐ecological contexts in which material practices may acquire reproductive advantages through their effects on coordination, affiliation, affective regulation, attention, expectation, and other forms of social organization. Such effects may contribute to survival and reproduction in broad and indirect ways, often through their influence on cooperation, exchange, mate choice, social buffering, or group stability. At the same time, the cultural success of a material practice depends not only on these broader consequences but also on the psychological biases discussed in (v–vi), which shape what individuals notice, value, remember, and copy. Cultural persistence, then, may depend on the interaction between three elements: recurrent coordination advantages, the phenomenal or affective features that make particular forms salient and compelling, and the social‐learning biases through which such forms are copied and retained under conditions of opacity. Consequently, the relation between social efficacy and cultural reproduction is often indirect: socially consequential practices may drift, persist, transform, or disappear depending on the transmission environments in which they are embedded Table 1.

**Table 1:** Three interacting levels in the reproduction of opaque social instruments.

Level	Role in the mechanism
Coordination pressures	Define the recurrent social problem‐space in which OSI‐like practices may acquire function
Social learning under opacity	Enables reproduction without explicit understanding through conformity, norm‐deference, and model‐based copying
Phenomenal and affective form	Makes particular variants salient, compelling, memorable, and therefore more likely to attract attention and copying

### 3.3 Transmission Dynamics Under Opacity

Identifying candidate social functions, phenomenal attractions, or learning biases does not by itself explain why a practice persists. Persistence typically requires a transmission pathway through which practices are reproduced with sufficient fidelity to remain socially effective across instances. This requirement is especially salient for OSIs, whose social efficacy typically exceeds users' explicit understanding. Explaining OSIs, therefore, requires not only specifying their coordination role but also accounting for how they are reproduced.

Three analytical layers can be distinguished. First, (i–iv) recurrent coordination pressures create adaptive space for OSI‐like practices, including through their effects on affective regulation. Second, (v) proximate learnability under opacity concerns how individuals acquire and reproduce practices without representing their underlying functions. This involves high‐fidelity copying of procedures through conformity, norm deference, and model‐based biases. In addition to this, (vi) certain formal or phenomenal features can increase the likelihood that particular variants are retained even when their social‐organizational role is not represented. Third, population‐level transmission dynamics determine how such practices become differentially copied across generations. In the present framework, transmission is not independent of social efficacy. Practices that successfully organize interaction are more likely to be reproduced, even when their coordinating function is not explicitly represented by participants.

While recurrent coordination pressures help explain why OSI‐like practices may become socially advantageous, the specific material forms they take are shaped by local transmission environments, understood here as the structured social conditions under which practices are learned and reproduced, including demographic structure, interaction networks, and cultural and institutional scaffolding. Once embedded in socially organized practices, variants may adapt to existing belief systems, aesthetic conventions, ecological constraints, or particular institutional settings, generating substantial cultural variation without altering the underlying coordination function.

Different transmission ecologies can be expected to generate different patterns of stability and variation. Some contexts might exhibit substantial formal continuity; others may show regional differentiation, clustering, or episodes of wider diffusion; still others may display bounded diversification. Multiple combinations of learning tendencies and demographic conditions can produce similar outcomes (e.g. [82, 83, 84, 85]), and archaeological patterning is therefore typically underdetermined at the level of specific psychological mechanisms. Related debates about the relationship between population size and cultural complexity (e.g. [86, 87, 88]) have highlighted both the importance of interaction networks and transmission structures and the difficulties of inferring their effects archeologically.

Still, different transmission ecologies constrain the range of possible patterning. Highly norm‐enforced environments are unlikely to produce unconstrained drift; highly segmented networks are unlikely to produce complete homogeneity. Such patterns do not depend solely on copying biases and demographics, but emerge from the interaction between recurrent coordination pressures, phenomenal and learning biases, and the transmission environments in which particular material practices become salient, copied, and retained.

The present framework, then, operates at the level of coordination pressures, phenomenal and affective salience, and reproduction dynamics, rather than at the level of specific cultural contents. It identifies the social, perceptual, and transmission‐related factors that make materially mediated practices likely to become socially useful, phenomenally compelling, copied, reproduced, and patterned across transmission ecologies. The distinction developed below between participation‐embedded and frame‐embedded forms captures one structured differentiation within this space, corresponding to different modes of social embedding and transmission.

## 4 A Basic Typology of Opaque Social Instruments (OSIs) for Archeology

“Symbolic” artifacts are often approached as sign‐objects whose primary explanatory burden is to carry meanings that must be decoded. The OSI framework shifts the explanatory starting point. It treats many canonical “symbolic” candidates instead as materially mediated configurations that become publicly recruitable within socially organized practices of recognition, affiliation, and role‐regulation, while typically being experienced by participants as customary, compelling, beautiful, sacred, or otherwise normatively loaded rather than as explicit tools of social organization. The central question is therefore not primarily what such artifacts meant, but how they became embedded in social practices through which they became efficacious under conditions of functional opacity.

Once “symbolic” artifacts are treated as OSIs in this sense, the question arises of how such practices should be analytically differentiated. The OSI framework is intended as a generative scaffold rather than a closed taxonomy, and different OSIs might be sorted along multiple axes, including function, scale, enforcement, and demographic reach. For Pleistocene archeology, however, one dimension proves especially tractable: the mode of social embedding through which an OSI becomes efficacious. These modes correspond to different kinds of transmission environments and social ecologies within which practices involving OSIs are reproduced.

### 4.1 Participation‐Embedded and Frame‐Embedded OSIs

This section distinguishes between PE‐OSIs and FE‐OSIs. This distinction is heuristic rather than taxonomic, and no hard boundaries are implied. Many material practices may occupy intermediate positions. The point is not to divide artifacts into mutually exclusive boxes, but to capture a broad yet salient contrast in how their coordinating functions are socially enacted and maintained, where “enacted” indicates that social significance is realized in embodied, socially structured activity rather than through representational decoding (e.g. [89, 90]).

PE‐OSIs are those whose social force derives primarily from recurrent embodied participation within interactionally dense settings – shared routines, skill display, and practical know‐how acquired through repeated co‐presence. Their intelligibility rests on shared perceptual and practical experience: ecological familiarity, embodied participation, skill recognition, everyday coordinated activity, and associative cues internal to a practice ecology. Repeated engagement in hunting, foraging, knapping, shell collecting, or pigment processing can make certain materials socially legible through the same learning biases and patterned interaction described earlier.

Early candidates plausibly include over‐determined handaxes (bifaces whose investment in symmetry, refinement, or finishing plausibly exceeds what is required for cutting performance), curated pigments, pendants, and early shell beads (e.g. [7, 9, 91, 92]). Also, a lion claw, for instance, may become socially salient in relation to hunting practice and the social evaluation of risk, competence, or prowess [93]. Such items become socially legible on the basis of shared practices (knapping, hunting) without requiring a cross‐generational interpretative framework.

By contrast, FE‐OSIs are those whose social force depends primarily on institutionally mediated transmission and publicly stabilized interpretive frameworks. Rather than relying mainly on practical familiarity acquired through repeated co‐presence, their efficacy presupposes transmission environments in which roles, expectations, placement rules, restrictions, or forms of instruction are stabilized beyond the immediate context of participation. Such environments may be encouraged by greater demographic scale, more extended interaction networks, and stronger institutional continuity across generations. The central difference is thus between participation‐dependent coordination and coordination stabilized through publicly maintained interpretive frames.

Although PE‐ and FE‐OSIs may support similar ultimate coordination functions, they emerge under different structural constraints. Participation‐embedded forms suffice where coordination is sustained through repeated co‐presence in interactionally dense settings. Frame‐embedded forms may become advantageous where coordination must extend across larger, more segmented, or temporally discontinuous populations. FE‐OSIs, therefore, do not reflect fundamentally new evolutionary pressures or a unilinear increase in cognitive or symbolic sophistication, but different transmission environments and pathways of social reproduction. The PE/FE distinction does not map directly onto geographic spread or diffusion, as participation‐embedded traditions may spread widely when their legibility derives from familiar practices or salient forms, while frame‐embedded traditions may remain highly localized if the interpretive framework does not travel.

Because the Pleistocene record rarely permits secure inferences about specific myths or worldviews, FE attributions must remain graded and evidentially conservative. They are strongest where material patterning exceeds what face‐to‐face participation alone would sustain. FE‐OSIs are therefore typically associated with spatially segregated and structured contexts, such as deep cave chambers, ritual installations, mortuary deposits, or architecturally delimited spaces, where access was selective or normatively regulated. These cases may show stronger evidence of curation, repair, and multi‐generational circulation, along with a more elaborate spatial “syntax,” including stable constellations, recurrent placement rules, and patterned associations with built features.

In this spirit, hybrid figurines and structured cave installations – such as the Hohlenstein‐Stadel Lion Man [94], the Brassempouy heads [95], or the Bruniquel stalagmite constructions [96] – can be discussed as candidates toward the FE end of the spectrum, insofar as their contexts suggest selective regulation, structured deployment, and a stabilizing framework not exhausted by craft skill or practical familiarity. Bruniquel, in particular, is relevant because it involves a Neanderthal construction with deliberate and patterned placement deep within a cave environment, features that raise the likelihood of access regulation and recurrent norms, while still leaving open what the practice “meant” in any semantic‐adjacent sense.

### 4.2 Applying the PE/FE Distinction

Table 2 does not function as a classificatory key for individual artifacts. It summarizes two modes of social embedding at the level of artifact–practice–context configurations. The relevant unit of analysis is therefore not the isolated object as encountered in a museum display case, but a reproducible pattern of making, using, placing, circulating, and recognizing material forms within a social ecology. Participation‐embedded forms are reproduced through recurrent embodied engagement in interactionally dense contexts, and frame‐embedded forms through publicly sustained interpretive frames that coordinate behavior beyond immediate co‐presence. In practice, of course, many cases lie along a continuum, and inference proceeds through the convergence of multiple lines of contextual evidence rather than through any single decisive criterion.

**Table 2:** A typology of participation‐embedded and frame‐embedded OSIs.

Dimension	Participation‐embedded social instruments (PE‐OSIs)	Frame‐embedded social instruments (FE‐OSIs)
Transmission environment	Reproduced within dense interaction networks where coordination is reproduced through recurrent embodied participation, shared routines, and practical skill recognition	Reproduced within transmission environments structured by publicly maintained interpretive frames that travel across contexts and generations (ritual templates, named roles, normative classifications, institutional scaffolds)
Mode of uptake	Learned primarily through practical familiarity and skill recognition acquired in embodied participation and repeated co‐presence	Learned through uptake of publicly stabilized roles, classifications, or normative expectations specifying how the item should be treated
Proximate experiential profile	Often experienced as skill‐linked, appropriate, attractive	Often experienced as normatively loaded, role‐defining, sacred/taboo, mythical, or standing for something
Functional opacity	Often medium to high: socially enacted without explicit representation of underlying system‐level coordination functions	Often high: frame‐rationalizations (mythic/normative explanations) can be orthogonal to system‐level functions
Archaeological indicators	Evidence of recurrent enactment within familiar practice settings: repair or maintenance, wear or use histories consistent with repeated participation in socially legible activities, and patterned clustering within everyday activity contexts. Assemblages may derive their social legibility from practices familiar within subsistence or craft activities (e.g., shells, animal remains, pigments)	Evidence consistent with frame‐mediated stabilization: patterned placement rules, durable associations with structured spaces or roles, selective access, and multi‐episode recurrence suggesting coordination not reducible to face‐to‐face participation alone. Candidate items may appear contextually distinctive or embedded within highly regulated settings requiring shared interpretive frameworks for their social recognition

Archeology frequently confronts us with singular, spectacular finds: a figurine, a structured installation, an unusual biface. Yet OSI‐attribution concerns whether such items plausibly instantiate a socially reproduced coordination configuration. A single object can be evidence for such a configuration insofar as it participates in a broader pattern: contextual clustering, structured placement, recurring production rules, circulation ranges, wear histories, or associations with other patterned practices. The question is not whether an object is “symbolic,” but whether the surrounding patterning indicates a participation‐dependent or frame‐embedded mode of coordination. As said, this does not exhaust the range of relevant inferences, which may also concern the specific coordination problems at stake, or whether they operate primarily in dyadic or wider group contexts.

For example, hand stencils are ubiquitous yet difficult to classify under icon/index/symbol frameworks, since they may simultaneously be described as iconic, indexical, and symbolic. Moreover, little follows from the observation that a stencil resembles a hand, refers to one, or indexes one. OSI analysis can instead ask: do stencils function primarily as participation‐embedded marks of co‐presence and inclusion in a shared practice (PE‐leaning), or do their placement rules, access constraints, and patterned associations suggest a more stabilized, trans‐contextual normative framing (FE‐leaning)? Either way, the explanatory focus shifts from decoding representational content to understanding how materially mediated practices stabilized coordination.

The same material form, then, may participate in both embedding modes. Ornament types such as beads may function as participation‐embedded markers of skill, attractiveness, or affiliation in some contexts, while in others they may be incorporated into frame‐embedded roles or ritualized exchanges. Because such distinctions concern the stabilization of practices rather than the morphology of artifacts, they cannot usually be inferred from objects in isolation. Nor does broad geographic distribution by itself license an inference to frame embedding. Instead, inference relies on contextual patterning that indicates whether or not coordination plausibly depended on more stabilized interpretive frameworks.

These expectations are operationalizable using familiar archaeological methods. The OSI framework thus operates with a logic many archeologists already employ implicitly, while redirecting attention toward social function, opacity, and transmission rather than representation and the decoding of meaning.

## 5 Conclusion

This paper has argued that prehistoric “symbolic” artifacts are often better understood not as symbolic signs, but as opaque social instruments: materially mediated coordination devices whose social effects typically exceed users' explicit understanding of their functions. Ornaments, pigments, figurines, and related items need not be approached primarily as vehicles of encoded meaning or as straightforward proxies for language‐like symbolic cognition. They can instead be understood as artifact–practice configurations whose reproduction was supported both by recurrent coordination advantages and by the phenomenal, affective, and social learning biases that rendered them salient, memorable, attractive, and readily copied. This reframing is not intended to displace existing interpretive approaches, but to provide a complementary explanatory lens focused on coordination and transmission under opacity.

The framework developed here offers a basic mechanistic account of how such practices may become reproducible and persist. It brought together three explanatory levels: recurrent coordination pressures that create adaptive space for OSI‐like practices; learning, copying, and content biases that make such practices highly reproducible without explicit understanding; and transmission environments that shape how materially mediated coordination practices persist and diversify across populations. On this view, prehistoric “symbolic” traditions become intelligible not through the postulation of hidden meanings, but through the interaction of coordination demands, phenomenal salience, social learning, and patterned transmission.

The distinction between participation‐embedded and FE‐OSIs extends this proposal by identifying two broad modes of social embedding within which such practices may become socially legible. The point is not to classify objects in isolation, nor to posit a unilinear progression from “simple” to “complex” symbolism, but to ask what kinds of social embedding and transmission environments would have been sufficient to reproduce the archaeological pattern observed. This shifts attention from artifact morphology alone to artifact–practice–context configurations, and from meaning‐decoding to the minimal conditions under which materially mediated coordination could be reproduced and sustained. It also leaves room for future work to refine the distinction or introduce different dimensions of variation.

For Paleolithic archeology, this reframing turns the vague category of “symbolism” into a more tractable set of questions. Rather than asking first what an artifact represented, analysts can ask what coordination problems it may have helped solve, how it was maintained or recurrently reproduced despite functional opacity, and what contextual patterning would be expected under different transmission environments. This provides an inferential framework for comparing sites, evaluating candidate interpretations, and deriving testable expectations about clustering, placement, curation, circulation, and other visible patterns.

More broadly, the OSI framework has implications beyond archeology. First, this reframing challenges the widespread inference from “symbolic” material culture to symbolic cognition: if symbolic‐looking practices can stabilize without shared semantic representation, then their presence does not by itself license inferences about language‐like thought, abstraction, or semantic encoding. This bears directly on debates about the origins of language and “modern behavior,” where the appearance of symbolic artifacts is routinely taken as evidence for representational capacities.

Second, it offers a way of linking debates on prehistoric symbolism to work in cultural evolution, evolutionary psychology, and social cognition by treating “symbolic” culture as a domain of cumulative cultural adaptation rather than as a sudden threshold in representational capacity. It suggests new routes for formal and comparative work, including transmission modeling, agent‐based simulations of transmission and reproduction under opacity, and even ethnographic or experimental studies comparing explicit interpretations of artifacts with their social‐organizational effects. If successful, such work would help relocate the study of early “symbolic” material culture from the search for hidden meanings to the analysis of the mechanisms through which socially functional, phenomenologically compelling, and often opaque practices emerged and endured.

## Data Availability

Data sharing is not applicable to this article as no datasets were generated or analyzed during the current study.
